# Systemic treatment of metastatic uveal melanoma: review of literature and future perspectives

**DOI:** 10.1002/cam4.133

**Published:** 2013-09-18

**Authors:** Kristina Buder, Anja Gesierich, Götz Gelbrich, Matthias Goebeler

**Affiliations:** 1 Department of Dermatology, Venereology and Allergology, University Hospital WürzburgJosef-Schneider-Strasse 2, Würzburg, 97080, Germany; 2 Comprehensive Cancer Center Mainfranken, University Hospital WürzburgJosef-Schneider-Strasse 6, Würzburg, 97080, Germany; 3 Institute for Clinical Epidemiology and Biometry, University of WürzburgJosef-Schneider-Straße 2, Würzburg, 97080, Germany

**Keywords:** Clinical trials, drug therapy, metastatic, review, uveal melanoma

## Abstract

Up to 50% of patients with uveal melanoma develop metastatic disease with poor prognosis. Regional, mainly liver-directed, therapies may induce limited tumor responses but do not improve overall survival. Response rates of metastatic uveal melanoma (MUM) to systemic chemotherapy are poor. Insights into the molecular biology of MUM recently led to investigation of new drugs. In this study, to compare response rates of systemic treatment for MUM we searched Pubmed/Web of Knowledge databases and ASCO website (1980–2013) for “metastatic/uveal/melanoma” and “melanoma/eye.” Forty studies (one case series, three phase I, five pilot, 22 nonrandomized, and two randomized phase II, one randomized phase III study, data of three expanded access programs, three retrospective studies) with 841 evaluable patients were included in the numeric outcome analysis. Complete or partial remissions were observed in 39/841 patients (overall response rate [ORR] 4.6%; 95% confidence intervals [CI] 3.3–6.3%), no responses were observed in 22/40 studies. Progression-free survival ranged from 1.8 to 7.2, median overall survival from 5.2 to 19.0 months as reported in 21/40 and 26/40 studies, respectively. Best responses were seen for chemoimmunotherapy (ORR 10.3%; 95% CI 4.8–18.7%) though mainly in first-line patients. Immunotherapy with ipilimumab, antiangiogenetic approaches, and kinase inhibitors have not yet proven to be superior to chemotherapy. MEK inhibitors are currently investigated in a phase II trial with promising preliminary data. Despite new insights into genetic and molecular background of MUM, satisfying systemic treatment approaches are currently lacking. Study results of innovative treatment strategies are urgently awaited.

Forty clinical studies on metastatic uveal melanoma were reviewed regarding responses to systemic treatments. New insights into genetic and molecular background led to investigation of new substances but promising in vitro data have not yet been translated into satisfying treatment responses; however, preliminary results of ongoing studies are highly encouraging.

## Introduction

Ocular melanoma accounts for 3% of all melanoma cases 1. Uveal melanoma (UM) is the most common primary intraocular tumor with an incidence of approximately five cases per million individuals 1. Up to 50% of patients develop metastatic disease with spread of tumor cells to liver (89%), lung (29%), bone (17%), and other organs 1,2. At this stage UM has a poor prognosis with median overall survival (OS) of 4–15 months 3. Survival rates in metastatic UM (MUM) have remained almost unchanged in the past 40 years 1.

As far as MUM is restricted to a limited anatomic region, locoregional treatment modalities can be used to control disease, for example, surgical resection, intraarterial chemotherapy, transarterial percutaneous chemoembolization, selective internal radiation therapy, and radiofrequency ablation 4. Patients in whom surgical resection is feasible show longer OS 5. Liver-directed therapies may induce remission of single metastases but do not prolong OS 4.

MUM is frequently treated with chemotherapeutics like dacarbazine, fotemustine, or gemcitabine/treosulfan although evidence for these regimens is limited. In clinical practice, responses are rarely seen and the impact of systemic chemotherapy on patients' survival is questionable 3.

Our understanding of molecular genetics and intracellular signaling pathways involved in the pathogenesis of MUM has improved over the last decades 6 resulting in the current investigation of targeted therapy approaches. We here review the present status of systemic treatment of MUM and evaluate therapy outcome measured by overall response rate (ORR) (IBM, Ehningen, Germany).

## Methods

PubMed search was performed for “metastatic” [and] “uveal” [and] “melanoma” as well as for “melanoma” [and] “eye” [and] “treatment” on 16 May 2013 for the time period between 1980 and May 2013. “Web of Knowledge” and congress abstract search via the American Society of Clinical Oncology homepage was performed (data cut 22 May 2013). The http://ClinicalTrials.gov website was searched for terms “melanoma” and “eye” on 13 May 2013. All retrieved study summaries were screened and compared to published data.

**Figure 1:**
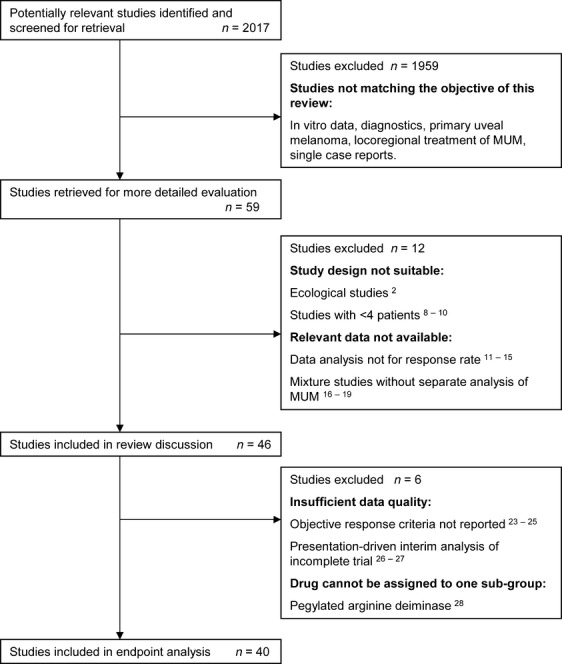
Flow of information through the different phases of the review process according to PRISMA statement 7.

All titles and abstracts in English language were screened for relevant content by the first author (K. B.). The selection process was documented according to PRISMA criteria (Fig. 1) 7. Studies on in vitro data, diagnostics, treatment of the primary tumor, single case reports, and clinical trials on locoregional treatment modalities were excluded. Full text versions of all relevant articles in English language were obtained and their references reviewed for additional relevant reports. Studies with less than four MUM patients, ecological design, without description of objective response assessment and studies not reporting ORR were excluded from meta-analysis (Fig. 1). All remaining studies were reviewed for quality aspects including study design, patient population, histological confirmation of disease, and method of staging/outcome evaluation by first and second author (K. B., A. G.). Patients treated in higher than first-line situations were classified as “non-first-line.”

Studies were grouped by type of treatment into single-agent or combination chemotherapies, chemoimmunotherapies, immunotherapies, antiangiogenetic therapies, and treatment with kinase inhibitors. In each group, rates of complete (CR) or partial remission (PR) and their exact 95% confidence intervals (95% CI) were computed for each study and overall for the group. In addition, homogeneity of ORR was examined by the exact chi-squared test. In case that homogeneity was rejected, the ORR was computed again, excluding the outlier study that caused heterogeneity. An overall summary analysis was carried out equally for all types of treatment. Statistical analysis was performed using SPSS statistics program version 21.0 (IBM, Ehningen, Germany).

## Results

The selection process is outlined in Figure 1. Of 59 retrieved articles including 11 congress abstracts, four were excluded because of small patient numbers (*n* < 4) 8–10 or ecological design 2. Nine were excluded because ORR was not reported 11–15 or mixture study design did not permit separate analysis of MUM data 16–19. Forty-six studies were included in review discussion, that is, one case series, five pilot, three phase I, one phase I/II, 29 phase II, and one phase III study, data from three expanded access programs, and four retrospective data analyses. In six of the studies response criteria were insufficiently described. The first authors of these reports were contacted by e-mail to comment on response criteria. In case of authors' response studies were included in numeric analysis 20–22 while studies for which response criteria could not be elucidated were excluded 23–25. Two publications were excluded because of presentation-driven interim analyses of incomplete clinical trials, one of them reported within a review publication 26,27 (NCT00338130, NCT01143402). One publication was excluded from numeric analysis because the drug could not be assigned to one treatment subgroup 28.

The numeric analysis included 40 publications with a total of 841 patients (Table 1). Patient numbers ranged from four in a pilot study 29 to 171 patients in a randomized multicenter study 30. Histological confirmation of metastatic disease was reported in 19/40 studies. Immunohistochemical stains of c-kit were performed in one study 31, mutational analysis of c-kit in another study 32 and GNAQ sequencing in a limited number of patients in two studies 33,34. Mean patients' age was 59 years; 546 patients were treated in first-line situation whereas 229 patients had received prior therapies including surgery, liver-directed treatment, chemotherapy, or immunotherapy. Response was evaluated according to WHO response criteria of 1979 35 in 12 and according to RECIST 1.0/1.1 36 in 27 studies.

**Table:** Study characteristics.

Author	Year	Drug	Study design	Response assessment	*n*	First-line	Non-first-line	Mean age	SD	PR/CR	ORR (%)	PFS (mon)	OS (mon)	Histology/genetics
Spagnolo	2013	Fotemustine	Retrospective	RECIST	24	24	0	62	9	2/0	8.3	unk	13.9	no/no
Leyvraz	2012	Fotemustine (iv vs. ia)	Phase III	RECIST	83 (a)	83	0	59	unk	2/unk	2.4	3.7	unk	yes/no
Homsi	2010	DHA-paclitaxel	Phase II	RECIST	22	11	11	56	7	1/0	4.6	3.0	9.8	no/no
Bedikian	2008	Liposomal vincristine	Pilot	WHO	4	unk	unk	56	unk	0/1 (b)	25.0	unk	unk	yes/no
Schmidt-Hieber	2004	Bendamustine	Phase II	RECIST	11	0	11	61	0	0/0	0.0	unk	unk	yes/no
Bedikian	2003	Temozolomide	Phase II	WHO	14	9	5	53	2	0/0	0.0	1.8	6.7	no/no
Ellerhorst	2002	Nitro-camptothecin	Phase II	WHO	14	0	14	59	2	0/0	0.0	unk	unk	no/no
Atzpodien	2008	Cisplatin (iv vs. ia)/gemcitabine/treosulfan	Pilot	WHO	12	1	11	62	6	0/0	0.0	unk	6.0	no/no
O'Neill	2006	Dacarbacine/treosulfan	Phase II	RECIST	14	15	0	64	2	0/0	0.0	3.0	7.5	no/no
Schmittel (a)	2005	Cisplatin/gemcitabine/treosulfan	Phase II	RECIST	17	19	0	60	7	0/0	0.0	3.0	7.7	yes/no
Flaherty	1998	Diverse chemotherapies	Retrospective pooled analysis	WHO	64 (c)	unk	unk	59	unk	5/1	9.0	unk	5.2	no/no
Sacco	2013	Dacarbazine	Phase II, randomized	RECIST	37	37	0	unk	4	3/unk	8.0	3.9	8.7	no/no
		Sunitinib			37	37	0	unk	9	0/0	0.0	2.8	6.4	
Schmittel	2006	Treosulfan	Phase II, randomized	RECIST	24	17	7	58	3	0/0	0.0	2.0	unk	yes/no
		Gemcitabine/treosulfan			24	15	9	63	7	0/1	4.2	3.0	unk	
Corrie	2005	Gemcitabine/treosulfan	Phase I	RECIST	5	4	1	50	4	0/0	0.0	6.8	13.3	yes/no
Schmittel (b)	2005	Gemcitabine/treosulfan	Phase II	RECIST	33	28	5	62	14	1/0	3.0	2.5	7.5	yes/no
Terheyden	2004	Gemcitabine/treosulfan	Phase II	WHO	20	8	14	62	5	0/0	0.0	unk	11.6	yes/no (d)
Keilholz	2004	Gemcitabine/treosulfan	Phase I	RECIST^1^	33	28	5	62	15	1/0	3.0	unk	unk	yes/no
Pföhler	2003	Gemcitabine/treosulfan	Pilot	WHO	14	13	1	63	8	3/1	28.6	7.1	15.3	no/no
Kivelä	2003	BOLD/INF-α2b	Phase II	WHO	22	24	0	61	2	0/0	0.0	1.9	10.6	yes/no
Pyrhönen	2002	BOLD/INF-α2b	Phase II	WHO	20	18	4	60	11	0/3	15.0	4.4	12.3	yes/no
Becker	2002	fotemustine/INF-α2b/IL-2	Phase II	WHO	25	unk	unk	56	unk	1/1	8.0	unk	15.0 (e)	no/no
Nathan	1997	BOLD/INFα-2b	Phase II	WHO	20	23	0	62	unk	4/0	20.0	unk	unk	yes/no
Kelderman	2013	Ipilimumab	EAP	RECIST, irRC	22	0	22	54	1	1/0	4.5	2.9	5.2	no/no
Khattak	2013	Ipilimumab	EAP	RECIST	5	0	5	42	2	0/0	0.0	unk	10.3	no/no
Danielli	2012	Ipilimumab	EAP	mWHO	9	0	13	57	2	0/0	0.0	unk	6.0	no/no
Khan	2012	Ipilimumab	Retrospective	RECIST, irRC	20	0	20	61	7	1/0	5.0	unk	unk	no/no
Piperno-Neumann	2013	Bevacizumab/temozolomide	Phase II	RECIST	35	35	0	55	9	0/0	0.0	3.0	12.0	no/no
Guenterberg	2011	Bevacizumab/INF-α2b	Phase II	RECIST	5	4	1	64	3	0/0	0.0	4.5	10.8	no/no
Tarhini	2011	Aflibercept	Phase II	RECIST	9	10	0	57	unk	0/0	0.0	5.7	19.0	yes/no
Zeldis	2009	Lenalidomide	Phase II	RECIST	16	unk	unk	53	7	0/0	0.0	unk	unk	no/no
Solti	2007	Thalidomide/INF-α2b	Pilot	RECIST	6	0	6	59	1	0/0	0.0	3.6	9.0	no/no
Reiriz	2004	Thalidomide	Phase II	WHO	5	0	5	59	1	0/0	0.0	unk	unk	yes/no
Bhatia	2012	Carboplatin/paclitaxel/sorafenib	Phase II	RECIST	24	20	4	61	12	0/0	0.0	4.0	11.0	no/no
Kaempgen	2012	Fotemustine/sorafenib	Case series	Investigator decision^1^	7	unk	unk	unk	unk	3/0	42.0	unk	unk	no/no
Falchook	2012	Trametinib	Phase I	RECIST	16	1	15	53	8 (f)	0/0	0.0	1.8	unk	yes/yes (f)
Kirkwood	2011	Selumetinib	Phase II	RECIST	7	20	0	57	unk	0/0	0.0	unk	unk	yes/yes (g)
Mahipal	2012	Sunitinib	Pilot	RECIST	18	3	17	69	12	1//0	5.0	4.2	8.2	no/no
Nathan	2012	Imatinib	Phase II	RECIST	25	24	13	63	unk	2/0 (h)	8.0	3.0	7.4	yes/yes (h)
Hofmann	2008	Imatinib	Phase II	RECIST	9	9	3	63	1	0/0	0.0	unk	6.8	yes/yes (i)
Penel	2008	Imatinib	Phase II	RECIST	10	6	7	58	1	0/0	0.0	unk	10.8	yes/no

a, only iv-group considered; b, CR patient had lung metastases only; c, 5/64 patients received chemotherapy/IL-2; d, not in all patients; e, OS includes intraarterially treated patients; f, no correlation of GNAQ status and response (GNAQ testing in six patients); g, GNAQ mutated in four, wild type in eight patients; h, c-kit exons 11, 13, and 17 analyzed, both PR patients c-kit wild type; i, c-kit immunohistology; bid, twice daily; EAP, expanded access program; iv, intravenous; ia, intraarterial (hepatic); mon, months; *n*, number of patients; unk, unknown; ORR, overall response rate; CR, rates of complete; PR, partial remission; PFS, progression-free survival; OS, overall survival.

First authors were contacted by email for clarification of objective response assessment.

Response, including CR and PR, was achieved in 39 of 841 patients; ORR was 4.6% (95% CI 3.3–6.3%). No responses were observed in 22/40 studies. Stable (SD) versus progressive disease (PD) was reported over all studies for 184 versus 379 patients (ratio 1:2) while nine studies did not provide information on SD/PD numbers. Median OS was reported in 26/40 studies ranging from 5.2 months in pretreated, predominantly end-stage patients 37 to 19.0 months in selected first-line patients 38. Progression-free survival (PFS) was reported in 21/40 studies ranging from 1.8 to 7.1 months.

**Figure 2:**
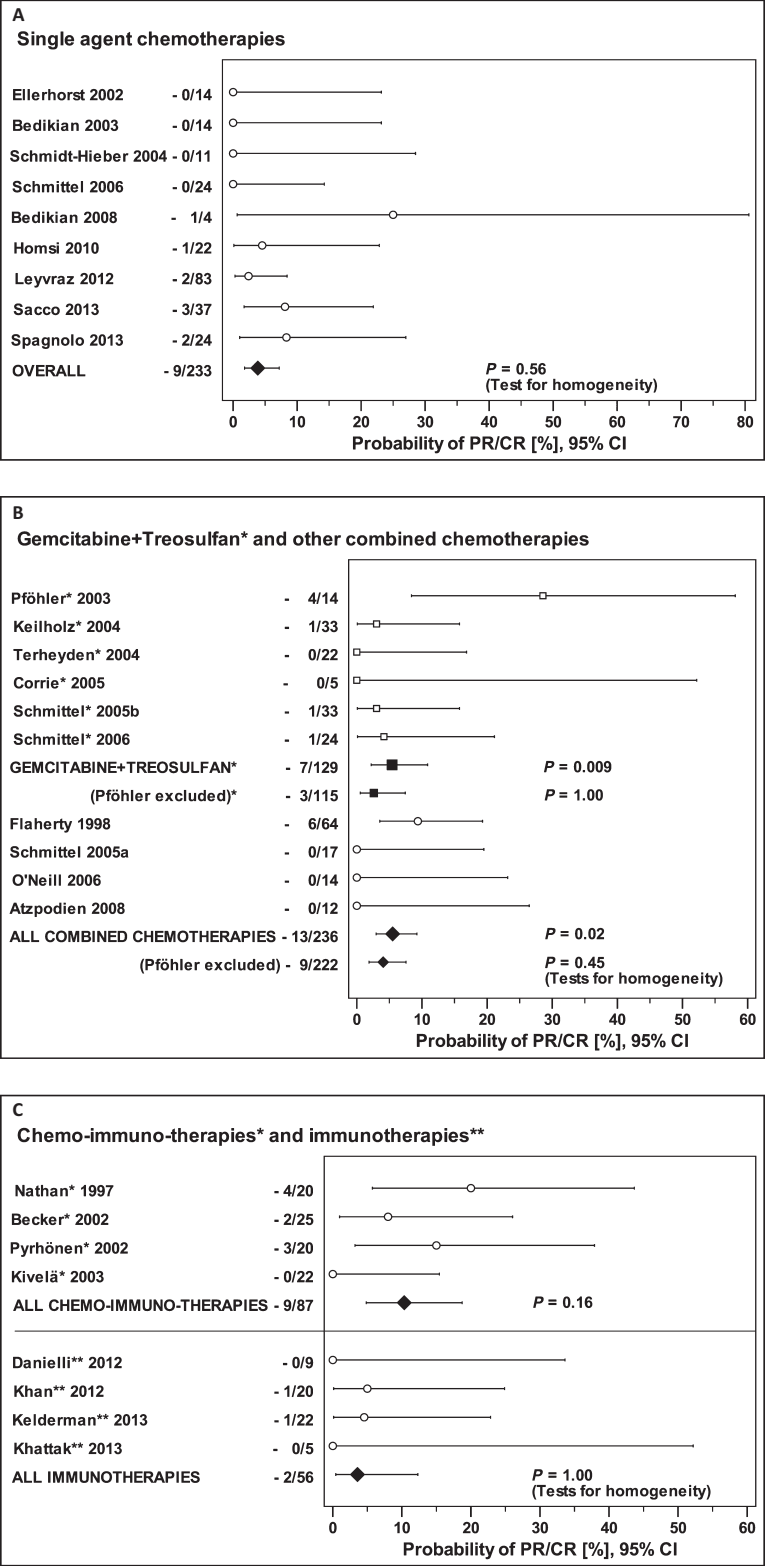
Response rates for single-agent chemotherapies (A), combination chemotherapies (B), and chemoimmunotherapies and immunotherapy with ipilimumab (C).

Single-agent chemotherapeutic regimens (dacarbazine 22, fotemustine 30,39, DHA-paclitaxel 40) showed ORR below 10% with the exception of a small pilot study (1 CR/4 patients) 29. Notably, four studies with smaller sample sizes observed no PR/CR (temozolomide 34,41, camptothecin 42, bendamustine 43, treosulfan 44). Testing for equal ORR did not detect substantial heterogeneity (*P* = 0.56). The estimated ORR was 3.9% (95% CI 1.8–7.2%) (Fig. 2A). Most of the patients were treated in non-first-line situations.

The best-investigated combination chemotherapy regimen is gemcitabine/treosulfan, tested in six phase I and II trials (Fig. 2B). An outstanding ORR of 28.6% (one CR, three PR in 14 patients) with OS of 15.3 months, and PFS of 7.1 month 45 could not be reproduced by subsequent studies on gemcitabine/treosulfan with more than 20 patients each and histology-proven disease in 4/5 studies 21,42,44–46. Reports on combination chemotherapies including cisplatin/gemcitabine/treosulfan 46,47, dacarbazine/treosulfan 48, and carboplatin/paclitaxel/sorafenib 49 showed no responses. Analysis of all combination chemotherapies excluding Pföhler et al. 45. for homogeneity reason revealed responses in 9/222 patients (ORR 4.1%; 95% CI 1.9–7.6%).

Chemoimmunotherapy regimens (bleomycin/vincristine/lomustine/dacarbazine [=BOLD]/INF-α2b, fotemustine/INF-α2b/IL-2) were studied in four phase II trials with 20–25 patients each, mainly in first-line situations with histology-proven MUM in 3/4 studies 50–53. The test for equal ORR did not detect substantial heterogeneity (*P* = 0.16); estimated ORR was 10.3% (95% CI: 4.8–18.7%) (Fig. 2C).

Ipilimumab immunotherapy (3 and 10 mg/kg) was analyzed in three expanded access programs and one retrospective single-center study in non-first-line situations 37,54–56. Two of 56 evaluable patients experienced PR (ORR 3.6%; 95% CI 0.4–12.3%) (Fig. 2C) while 12 patients showed disease stabilization.

**Figure 3:**
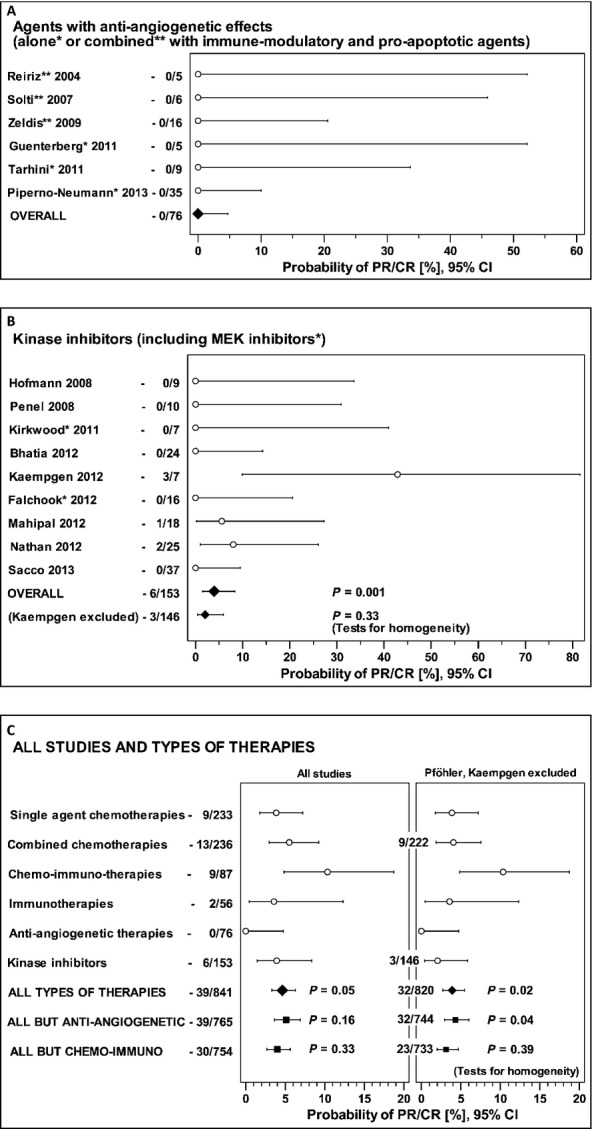
Response rates for agents with antiangiogenetic effect (A), kinase inhibitors (B), and comparison of all treatment modalities (C).

Antiangiogenetic treatment strategies using bevacizumab combined with interferon-α2b 57, temozolomide 58, or the vascular endothelial growth factor (VEGF)-trap aflibercept 38 did not show responses in first-line treatment. The antineoplastic and antiangiogenetic drug thalidomide failed to show responses in second-line situations as single agent 59 and in combination with interferon-α2b 60. Moreover, lenalidomide, which has antiangiogenetic and immunomodulatory properties, did not induce responses 61. Altogether, in 56 evaluable patients ORR was 0% (95% CI 0–4.7%) (Fig. 3A).

Recent study protocols focus on small molecule kinase inhibitors for targeted therapy of MUM (Fig. 3B). In three studies, imatinib (targets c-kit, platelet-derived growth factor [PDGF]) was applied as first- or second-line treatment (300 or 400 mg bid); 2/3 studies showed no responses 31,62. Two PRs (8%) were observed in one study with 25 patients; both responders presented c-kit wild-type status in the assessed metastases 32. Sunitinib (targets PDGF receptor [PDGFR], VEGF receptor [VEGFR], c-kit, and others) was studied in a pilot trial mainly in second-line situations. One PR (1/18, ORR 5%) and a relatively high proportion of patients in SD status (12/18) were reported 63. Sunitinib was therefore compared to dacarbazine in a randomized phase II trial that revealed no response in the sunitinib (0/37) versus 3 responses in the dacarbazine group (3/37). PFS was not improved in the sunitinib group. Sorafenib (targets RAF, VEGFR, c-kit, PDGFR) was investigated as single agent and in combination with chemotherapy. In a mainly first-line setting sorafenib failed to induce response but 12 of 24 patients showed SD 49. Phase I/II trials on mitogen-activated protein kinase (MEK) inhibitors selumetinib and trametinib that altogether recruited 23 MUM patients showed no responses 33,34. Falchook et al. 33 observed SD in 8/16 second-line patients (50%) with SD achievement not correlating with the mutational status. Overall, kinase inhibitors showed responses in 3/146 patients (ORR 2.1%; (95% CI 0.4–5.9%) (Fig. 3B).

### Future perspectives

Advances in knowledge about genetics and signaling pathways led to initiation of clinical trials with innovative therapeutics. Screening the http://ClinicalTrials.gov website for ongoing clinical trials on MUM revealed 15 studies, two of them with randomized design (Table 2). Only two of the phase II studies evaluate chemotherapies (albumin-bound paclitaxel 25, liposomal vincristine 29).

**Table:** Active/recruiting trials studying treatment approaches for metastatic uveal melanoma as registered on ClinicalTrials.gov.

ClinicalTrials.gov identifier	Drug	Phase	Planned patients	Status	Sponsor
NCT01355120	Ipilimumab (anti-CTLA4 antibody)	II	41	Data collection ongoing	University Hospital Essen, Germany
NCT01034787	CP-675,206 (anti-CTLA4 antibody)	II	32	Data collection ongoing	Alberta Health Services, Canada
NCT01585194	Ipilimumab (anti-CTLA4 antibody)	II	141	Recruiting	MD Anderson Cancer Center, US
NCT01587352	Vorinostat (histone deacetylase inhibitor)	II	32	Recruiting	National Cancer Institute, US
NCT01413191	Cixutumumab (anti-IGF-1R antibody)	II	32	Data collection ongoing	National Cancer Institute, US
NCT01200342	Genasense/oblimersen (Bcl-2 antisense oligonucleotide) plus carboplatin/paclitaxel	II	30	Data collection ongoing	MD Anderson Cancer Center, US
NCT00506142	Marqibo (liposomal vincristine)	II	50	Recruiting	Talon Therapeutics, US
NCT00738361	Abraxane (nab-paclitaxel)	II	25	Completed, results pending	National Comprehensive Cancer Network, Ohio, US
NCT01252251	Everolimus (mTOR inhibitor) and pasireotide (somatostatin receptor analog)	II	25	Recruiting	Memorial Sloan-Kettering Cancer Center, US
NCT01200238	Ganetespib (HSP90 inhibitor)	II	30	Recruiting	Dana-Farber Cancer Institute, US
NCT01143402	Selumetinib (MEK inhibitor) versus temozolomide	II	159	Recruiting	National Cancer Institute, US
NCT01430416	AEB071 (protein kinase C inhibitor)	I	65	Recruiting	Novartis Pharmaceuticals, US
NCT01377025	Sorafenib versus placebo	II	200	Recruiting	University Hospital, Essen, Germany
NCT01801358	AEB071 (protein kinase C inhibitor) plus MEK162	I/II	90	Not yet recruiting	Novartis Pharmaceuticals, US
NCT01835145	Cabozantinib versus temozolomide or dacarbazine	II	69	Not yet recruiting	National Cancer Institute, US

The occurrence of UMs in an immunologically privileged site makes immunotherapy a promising treatment approach. Current data on ipilimumab were gained from retrospective analyses only. One was published at the time of manuscript revision and showed, in line with the previously published studies, an ORR of 5.1% (2/39); SD was observed in 44% (week 12) and 25% (week 23) of patients 64. Anti-CTLA4 antibodies are further assessed in three prospective trials. While two of them are expected to report outcomes soon, another large trial on ipilimumab will not be finished before 2017 (Table 2). PD-1 and PD-L1 have become important targets in cutaneous melanoma. To our knowledge, MUM patients have not been included in trials with PD-1 or PD-L1 antibodies yet. However, as PD-L1 expression is found in MUM cells 65 and probably in the tumor environment further investigation of treatment strategies targeting PD-1/PD-L1 in MUM are warranted.

Activating somatic mutations in GNAQ/GNA11, two members of the guanine nucleotide-binding protein family (G-proteins), were found in 83% of UMs 66. Both mutations result in the constitutive activation of the mitogen-activated protein kinase (MAPK) pathway thereby inducing proliferation in the absence of external growth stimuli 67. Hence, blocking this pathway by specific inhibitors may be an effective therapeutic approach for MUM 68–70. Several kinase inhibitors are currently studied in five phase I/II and II studies. A phase II study presently conducted in the US compares selumetinib versus temozolomide/DTIC with a much noticed interim analysis on PFS; ORR was 15% in the selumetinib-group (7/46) compared to 0% in the temozolomide-group (0/46) and 0% in the cross-over group 26. However, tumor regression without reaching RECIST-defined response was seen in 50% in the selumetinib-group versus 11% in the temozolomide group and 23% in the cross-over group. PFS in week 16 was 43.1% for selumetinib versus 8.5% for temozolomide. Interestingly, responses were also seen in GNAQ/GNA11 (Q209, exon 5) wild-type patients. However, retrospective assessment of codon R183, exon 4 revealed a mutation in the patient with objective response according to RECIST. These promising but preliminary data on MEK inhibition had to be excluded from our numeric analysis as final study outcomes should be awaited 26,27.

GNAQ/GNA11 signaling induces activation of phospholipase C (PLC) and protein kinase C (PKC) further downstream of PLC with subsequent MAPK pathway activation 71. There are two trials under way investigating PKC inhibition alone and in combination with MEK inhibition. GNAQ/GNA11 signaling is also linked to the PI3K-AKT pathway in UM, usually in an activating manner resulting in increased cell proliferation and survival 71. Hence inhibition of PI3K or AKT, possibly in combination with MAPK pathway inhibition, appears to be another attractive treatment strategy.

On the basis of promising data on the multikinase inhibitor sorafenib in small case series, a placebo-controlled phase II study is currently conducted in Germany investigating sorafenib versus placebo. Preliminary data on cabozantinib, a c-Met/VEGFR2 inhibitor currently under investigation 12, prompted investigators to initiate a randomized phase II study on cabozantinib versus dacarbacine or temozolomide. Search on the http://ClinicalTrials.gov website additionally revealed results on one terminated, yet unpublished study analyzing the combination of sunitinib/lenalidomide/cyclophosphamide, which showed no response in 12 patients (NCT00482911).

Mutations in BAP1, a deubiquitinating enzyme located on chromosome 3p, are seen in 85% of high-risk (“class-2”) UMs and correlate with development of metastatic disease 72. One substrate of BAP1 is histone H2A; histone-deacetylase inhibitors were shown to reverse the H2A hyperubiquitination caused by BAP1 knock-down in vitro 73 and might therefore be a therapeutic strategy 74. The histone-deacetylase inhibitor vorinostat is currently studied in MUM.

Antiapoptotic bcl-2, which is (over)expressed in more than 95% of UMs 72, provides another potential target. The bcl-2-antisense oligonucleotide oblimersen is currently under investigation. Upregulation of insulin-like growth factor (IGF)-1 and IGF-1R receptor in UM 72 potentially offers the possibility of treatment with the anti-IGF-1R-antibody cixutumumab. Further compounds currently under investigation in phase II studies include the HSP90 inhibitor ganetespib, and the somatostatin receptor analog pasireotide in combination with everolimus. Other treatment approaches such as targeting of somatostatin receptors by octreotid 24 and a phase I/II study on pegylated arginine were disappointing 28.

Altogether, immunotherapeutics and kinase inhibitors are currently the most investigated agents with encouraging interim results on MEK inhibition.

## Discussion

Depending on the genetic signatures of the primary tumor 6, up to 50% of UM patients develop metastatic disease. Once metastases occur prognosis is bad and therapeutic options are limited with ORR being considerably low.

The only randomized controlled phase III trial on treatment of MUM (intravenous vs. intraarterial fotemustine) showed improved ORR of liver metastases and prolonged PFS in intraarterially treated patients but similar OS in both groups 30. Response to intravenous fotemustine was as low as 2.4%. Only two phase II studies have up to now been published that were designed as randomized trials with two subgroups 22,44. One of these showed 8% ORR in the dacarbazine group. Relatively high ORRs reported for single-agent or combination chemotherapy in small studies 29 are possibly due to selection bias in small patient numbers. Phase II trials on liposomal vincristine and albumin-bound paclitaxel are ongoing but uncertain to reproduce promising results of previous smaller studies 29,40.

In our pooled analysis, chemoimmunotherapy shows slightly better tumor responses than chemotherapy. This observation has to be interpreted with caution as our analysis considered first-line and higher line studies as well as studies that did not differentiate the outcome of first- and second-line treated patients. Better OS in the chemoimmunotherapy studies might thus partially be due to a first-line treatment situation in the majority of trials.

New insights into tumor biology led to investigation of immunotherapies, antiangiogenetic agents, and targeted therapies. While ipilimumab is effective in metastatic cutaneous melanoma 75, it did not yet appear to be superior to chemotherapy regimens in MUM, possibly because published data have mainly been generated from expanded access programs in non-first-line situations. However, OS of 5.2–10.3 months in pretreated patients might still be promising 37,54,56. Final conclusions can only be drawn from randomized studies, preferably in first-line situations, which are still lacking.

Although VEGF plays a major role in MUM 6, treatment regimens focusing on antiangiogenetic agents did not reveal responses in first-line treatment. Nevertheless, pooled OS of 12.7 months appear promising.

Kinase inhibitors including sorafenib, sunitinib, and imatinib did not show any responses in six of nine studies. Promising results from a small case series on sorafenib combined with fotemustine 20 led to initiation of a large phase II study of sorafenib the results of which are still pending. The ORR for sunitinib was 5% in a pilot trial 63, which, however, could not be confirmed in a subsequent randomized phase II study 22.

GNAQ/GNA11 mutations in over 80% of MUM leading to aberrant activation of the MAPK pathway especially makes MEK an attractive therapeutic target 6. Patients recruited in phase I/II studies, however, did not show objective responses upon MEK inhibitor treatment 33,34. Falchook et al. 33 did not observe a correlation between the mutational status of GNAQ/GNA11 and clinical response to MEK inhibition but the analyzed exons were not specified in the publication. A phase II study is currently conducted on selumetinib with a promising interim analysis but pending final results 26. According to preliminary data, there is no proven correlation of ORR or PFS with GNAQ/GNA11 mutational status. OS was not significantly improved compared to chemotherapy.

Increasing insight into the biology of MUM has not yet translated into higher ORR. Unexpectedly, a correlation of treatment response to mutational/expression status of molecular targets has not been found in smaller trials 32–34 and ongoing clinical studies 26. So far, there is no evidence of a clinically meaningful survival benefit due to novel targeted agents.

With respect to appallingly low ORR, the question is whether disease stabilizations are treatment related or simply reflecting the natural course of disease 13. None of the currently available therapies has shown prolongation of patients' OS. Survival data were reported in 75% of the analyzed studies but cannot be compared due to inhomogeneous patients' characteristics throughout the studies. Only 7/40 publications reported the lengths of metastases-free intervals as primary diagnosis of UM and, if reported, a wide range was seen within and among these studies (0–25 years) 39,49,50,53,63,76,77. As metastases may develop 10 or more years after primary UM, this “dormancy” phenomenon has a high impact on patients' prognosis 78,79. Moreover, other prognostic parameters such as lactate dehydrogenase, sites of metastases, and patients' performance status would need to be equally distributed in the studies to allow comparison of survival data.

According to available study data, survival appears to depend on patient- and tumor-related characteristics rather than on the actual treatment 3; it therefore can only be analyzed in randomized studies recruiting patients with comparable characteristics. Given a poor response rate in most of the studies, determining PFS at a certain time point might be a more suitable endpoint. This would require defined staging intervals, which, however, were inhomogeneous throughout the analyzed studies here and therefore not considered in this review.

## Conclusion

This review analyzes data of studies on systemic treatment of MUM published between January 1980 and May 2013. Altogether, published data mainly provided low-level evidence. The limited efficacy of current treatment approaches illustrates the high medical need for more effective treatment options in MUM.

To date, no chemotherapeutic, immunotherapeutic, or targeted drug has shown reproducible ORR >10% or prolonged OS in MUM. Targeted therapeutics as well as immunotherapies might be promising strategies, but need evaluation in prospective trials. Investigation of chemoimmunotherapy-based strategies appeared to become less important, probably due to toxicity profiles although ORR has been superior to all other therapeutic approaches. Most promising preliminary data are available for MEK inhibition. However, these therapeutic regimens should be judged after final data analyses become available. A future goal should be careful design of randomized clinical trials.

## Conflict of Interest

K. Buder received educational/travel grants and honoraria for oral presentations from TEVA GmbH, Roche Pharma, and Bristol-Myers Squibb. A. Gesierich received travel grants for congress participation, and was an advisory board member for Bristol-Myers Squibb and Roche Pharma. No conflicts of interest declared for G. Gelbrich. M. Goebeler was an advisory board member for MSD SHARP and DOHME GmbH.
